# Structural white matter networks in myotonic dystrophy type 1

**DOI:** 10.1016/j.nicl.2018.101615

**Published:** 2018-11-28

**Authors:** Maud van Dorst, Kees Okkersen, Roy P.C. Kessels, Frederick J.A. Meijer, Darren G. Monckton, Baziel G.M. van Engelen, Anil M. Tuladhar, Joost Raaphorst, for the OPTIMISTIC consortium

**Affiliations:** a Department of Medical Psychology, Radboud University Medical Center, Geert Grooteplein Zuid 10, Nijmegen 6525 GA, the Netherlands; b Department of Neurology, Donders Institute for Brain, Cognition and Behaviour, Radboud University Medical Center, Reinier Postlaan 4, 6525 GC, Nijmegen; c Department of Neuropsychology and Rehabilitation Psychology, Donders Institute for Brain, Cognition and Behaviour, Radboud University, Montessorilaan 3, Nijmegen 6525 HR, the Netherlands; d Department of Radiology and Nuclear Medicine, Radboud University Medical Center, Geert Grooteplein Zuid 10, Nijmegen 6525 GA, the Netherlands; e Institute of Molecular, Cell and Systems Biology, College of Medical, Veterinary and Life Sciences, University of Glasgow, Davidson BuildingUniversity Avenue, Glasgow G12 8QQ, UK; f Vincent van Gogh Institute of Psychiatry, Stationsweg 46, 5803 AC Venray, the Netherlands; g Department of Neurology, Amsterdam Neuroscience Institute, Amsterdam University Medical Center, Meibergdreef 9, Amsterdam 1105 AZ, the Netherlands

**Keywords:** Myotonic dystrophy type 1, MRI, White matter, Diffusion tensor imaging, Networks

## Abstract

The myriad of neuropsychiatric manifestations reported in myotonic dystrophy type 1 may have its origin in alterations of complex brain network interactions at the structural level. In this study, we tested the hypothesis that altered white matter microstructural integrity and network organisation were present in a cohort of individuals with DM1 compared to unaffected controls, which was expected to be associated with CNS related disease manifestations of DM1. We performed a cross-sectional neuropsychological assessment and brain MRI in 25 myotonic dystrophy type 1 (DM1) patients and 26 age, sex and educational level matched unaffected controls. Patients were recruited from the Dutch cohort of the OPTIMISTIC study, a concluded trial which had included ambulant, genetically confirmed DM1 patients who were severely fatigued. We applied graph theoretical analysis on structural networks derived from diffusion tensor imaging (DTI) data and deterministic tractography to determine global and local network properties and performed group-wise comparisons. Furthermore, we analysed the following variables from structural MRI imaging: semi-quantitative white matter hyperintensity load andwhite matter tract integrity using tract-based spatial statistics (TBSS). Structural white matter networks in DM1 were characterised by reduced global efficiency, local efficiency and strength, while the network density was compatible to controls. Other findings included increased white matter hyperintensity load, and diffuse alterations of white matter microstructure in projection, association and commissural fibres. DTI and network measures were associated (partial correlations coefficients ranging from 0.46 to 0.55) with attention (d2 Test), motor skill (Purdue Pegboard test) and visual-constructional ability and memory (copy subtest of the Rey-Osterrieth Complex Figure Test). DTI and network measures were not associated with clinical measures of fatigue (checklist individual strength, fatigue subscale) or apathy (apathy evaluation scale – clinician version). In conclusion, our study supports the view of brain involvement in DM1 as a complex network disorder, characterised by white matter network alterations that may have relevant neuropsychological correlations. This work was supported by the European Community's Seventh Framework Programme (FP7/2007–2013; grant agreement n° 305,697) and the Marigold Foundation.

## Highlights

• Structural white matter networks in DM1 display diffuse alterations in connectivity in comparison with unaffected controls
• Networks are characterised by decreased strenght, local and global efficiency, at a comparable density
• In this study, network parameters were slightly associated with measures of neuropsychological performance

## 1 Introduction

Myotonic dystrophy type 1 (DM1) is an hereditary chronic progressive multisystem disorder with autosomal dominant inheritance.(Bird, 1993) Clinical features of central nervous system (CNS) involvement in DM1 include cognitive deficits, psychiatric and behaviour disturbances such as apathy, fatigue, and excessive daytime sleepiness. These CNS symptoms are critical determinants of quality of life in DM1, and some may be amenable to treatment. (Antonini et al., 2006; Gagnon et al., 2008; Laberge et al., 2013; Okkersen et al., 2018) The broad spectrum of clinical CNS involvement in DM1 is corroborated by a variety of structural brain imaging abnormalities that are widely dispersed throughout the brain, with apparently little anatomical specificity.(Cabada et al., 2017; Okkersen et al., 2017b; Sugiyama et al., 2017) In particular, white matter involvement encompasses a combination increased white matter hyperintensity load and decreased microstructural integrity of white matter based on diffusion tensor imaging (DTI) studies.(Minnerop et al., 2011; Zanigni et al., 2016)

Given the exceptionally large clinical variability of the disease, in which the genetic defect exerts heterogeneous downstream effects in virtually all cells of many different tissue types, these diverse and widely dispersed structural imaging changes may come as no surprise.(Harper, 2001) Despite the many structural imaging changes, attempts at correlation with neuropsychological performance and other CNS features have given inconsistent results.(Cabada et al., 2017; Minnerop et al., 2011) Partly, this is explained by the variability of the disease in combination with limited sample sizes.(Okkersen et al., 2017a) Moreover, cognitive and behavioural functions are not strictly anatomically localised in particular brain regions, but have their origin in complex network interactions.(Bressler and Menon, 2010; Wang et al., 2015) In this respect, structural network analysis of white matter changes may improve understanding of brain dysfunction in complex neurological disorders with diffuse structural alterations, such as DM1.(Stam, 2014) Structural connectivity of a network, consisting of brain regions (nodes) and connecting white matter tracts (edges), can be obtained using analysis of DTI followed by tractography.(Yan et al., 2011; Zalesky et al., 2011) Graph theory, a branch of modern network theory, can subsequently be used to characterise the properties of the network organisation.(Bullmore and Sporns, 2009) Previous research in diseases that, like DM1, also have prominent white matter involvement (i.e., cerebral small-vessel disease) showed that white matter network alterations were an independent predictor of cognitive dysfunction and a more sensitive measure than traditional magnetic resonance imaging (MRI) measures.(Lawrence et al., 2014; Tuladhar et al., 2016) In this study, we tested the hypothesis that altered white matter microstructural integrity and network organisation was present in a cohort of individuals with DM1 compared to unaffected controls, which was expected to be associated with CNS related disease manifestations of DM1.

## 2 Materials and methods

### 2.1 Participants

We performed a single-centre, cross-sectional study. Patients were recruited from the Nijmegen subcohort (*n* = 66) of the Observational Prolonged Trial In Myotonic Dystrophy type 1 to Improve Quality of Life- Standards, a Target Identification Collaboration (OPTIMISTIC) study, a randomised trial in DM1 that investigated the effect of cognitive behavioural therapy with optional graded exercise on capacity for activity and participation.(Okkersen et al., 2018) This study recruited adult, severely fatigued (as defined by a checklist individual strength –fatigue subscale (CIS-fatigue) score ≥ 35) patients that were able to walk independently and had a genetically confirmed diagnosis of DM1; details have been published elsewhere.(Okkersen et al., 2018; van Engelen and Consortium, 2015). We invited patients to participate in the current sub-study, only after they had finished participation in the OPTIMISTIC main study. Specifically for this sub-study, we recruited 26 age-, sex- and educational level matched, unaffected, healthy, controls from the social network of DM1 participants. We excluded relatives unless they had been genetically tested for DM1 and the results of testing were negative.We selected controls from the social network to obtain a good match in terms of intelligence and social situation. Controls were not evaluated for apathy, depression or fatigue with questionnaires. This study was conducted in accordance with the provisions of the Declaration of Helsinki and Good Clinical Practice guidelines and the regional review board (CMO Arnhem-Nijmegen) of our institution approved the study. All participants provided prior written informed consent.

### 2.2 Study overview and eligibility criteria

Assessment visits took place over two separate days for each participant to reduce the influence of fatigue on (neuropsychological) tests. On the first day, the following variables were recorded: age, sex, educational level according to the Dutch educational system (Duits A. Kessels R., 2014) (7-point ordinal scale; 1 = less than primary school, 7 = academic degree). We excluded participants with significant known co-morbidities of the central nervous system that could interfere with neuropsychological or MRI-related outcomes by careful history taking. In addition, we excluded significant (nocturnal) hypoventilation by structured history taking for relevant symptoms (e.g. morning headaches, nightmares, non-revitalizing sleep) and by performing spirometry in supine and sitting position (patients were excluded if functional vital capacity (FVC) showed a difference of ≥20% between supine (lower value) and sitting position (higher value)), or a FVC <60% of expected in sitting position. Patients with known nocturnal hypoventilation who were stable on non-invasive ventilation were eligible for the study. A family history was taken to exclude any hereditary CNS diseases other than DM1. Following these procedures, participants underwent brain MRI. On the second day, a neuropsychological assessment was administered.

For further characterisation of our DM1 patient cohort, we utilised clinical information previously recorded in the OPTIMISTIC study: apathy (apathy evaluations scale, clinician version (AES-C)), capacity for activity and social participation (DM1-Activ-c), overall disease impact (myotonic dystrophy health index: MDHI), experienced fatigue (checklist individual strength fatigue subscale: CIS-fatigue) and muscular impairment (muscular impairment rating scale: MIRS) (Okkersen et al., 2018). In addition, we recorded the disease classification based on the age at onset of symptoms recorded in the OPTIMISTIC study (De Antonio et al., 2016).

### 2.3 Neuropsychological assessment and analyses

A single neuropsychologist (MvD) administered the neuropsychological assessment for each participant, in a neutral low-stimulus environment. The assessment took approximately 90 min. We selected tests to minimize the influence of motor response as much as possible and to evaluate cognitive function in seven different cognitive domains: abstract reasoning, memory, executive functioning, fluency, attention, visuospatial constructional abilities and psychomotor skill. We estimated premorbid intelligence with the Dutch version of the National Adult Reading Test (NART); abstract reasoning was evaluated with the 12-item short form of the Raven Progressive Matrices (RPM). Episodic memory was evaluated with the Wechsler Memory Scale – Fourth Edition (WMS-IV), subtests Logical Memory I and II (LMI and II); executive functioning was evaluated with the Trail Making Test (TMT), the Stroop Color Word Test and the Brixton Spatial Anticipation Test. We evaluated verbal fluency with the category fluency test (1-min animal and occupation naming). We assessed attention using the d2 Test by means of the total score on a subtest which corrected for the number of incorrect answers. The Rey-Osterrieth Complex Figure Test(ROCF) was used to evaluate visuospatial constructional abilities and visuospatial memory.(Shin et al., 2006) Finally, we administered the Purdue Pegboard Test as a measure of psychomotor skill.(Tiffin and Asher, 1948) For the Pegboard Test, we calculated the mean score of the left and right hand combined. We corrected the scores of some neuropsychological tests to minimize the effects of deficits in motor skill and dysarthria; we used these scores in subsequent between-group and correlation analyses. For the TMT, we recorded the response time and the number of correct answers on parts A and B and for each part calculated a accuracy-speed trade-off score by dividing the accuracy by the response time.(Schaapsmeerders et al., 2013) Then, we calculated the TMT interference score: the accuracy-speed trade-off for TMT B divided by the speed-accuracy trade-off for TMT-A.(Oosterman et al., 2010) A similar procedure was followed for the Stroop cards I, II and III. The Stroop interference score was calculated by dividing the accuracy-speed trade-off for Stroop card III by the accuracy-speed trade-off for Stroop Card II.(Raaphorst et al., 2011) Both interference scores were used in the analyses to measure executive function. More detailed information about the neuropsychological tests can be found in Lezak (2012).(Lezak, 2012)

### 2.4 MR imaging and analyses

#### 2.4.1 MRI acquisition

MRI scans were acquired on a Siemens Magnetom TRIO 3 T scanner (TIM trio, Siemens Medical Solutions, Erlangen, Germany). The protocol included a T1-weighted 3D magnetization-prepared rapid gradient-echo (MPRAGE) imaging (TR/TE = 1900/2.52 ms, flip angle 9°; voxel size 1.0 × 1.0 × 1.0 mm, FOV = 256 mm), a T2 TSE (TR/TE = 3830/125 ms, flip angle = 120°, voxel size 0.6 × 0.6 × 3 mm, FOV = 240 mm), a fluid-attenuated inversion recovery (FLAIR) sequence (TR/TE = 9000/86 ms, flip angle = 150°, voxel size 0.7 × 0.6 × 5 mm, FOV = 240 mm), and a Diffusion Tensor Imaging (DTI) single-shot spin-echo EPI, *b* values 0 and 1000 s/mm^2^, TR/TE = 7400/71 ms, number of encoding directions = 64, FOV = 240 mm, and voxel size 2 × 2 × 2 mm and 8 *b*0 unweighted images. All patients were scanned on the same MR scanner.

#### 2.4.2 White matter hyperintensities

White matter hyperintensities (WMH) were evaluated semi-quantitatively by an experienced neuroradiologist (F.M.) blinded for participant information. We defined a WMH as hyperintense signal on FLAIR images with no or slight hypointensity on T1 weighted images. WMH were scored using Fazekas' and age-related white matter changes scales (ARWMC) and were determined for periventricular, deep and basal ganglia white matter.(Fazekas et al., 1987; Wahlund et al., 2001).

#### 2.4.3 DTI processing

First, we denoised the raw diffusion weighted images for each participant using the Local Principal Component Analyses filter. We then preprocessed the diffusion images using an in-house developed Patching ArTefacts from Cardiac and Head motion (PATCH) algorithm to correct for artefacts resulting from cardiac artefacts, head motion, and eddy currents.(Zwiers, 2010) To eliminate echo planar imaging (EPI) distortions, the EPI images were normalized to T1-images only in the phase encoding direction.

We then applied DTIFIT from FSL to estimate the diffusion tensor and basic DTI parameters, including fractional anisotropy (FA), mean diffusivity (MD), axonal diffusivity (AD) and radial diffusivity (RD). The estimated DTI parameters were fed into the tract-based spatial statistics (TBSS) pipeline.(Smith et al., 2006) In short, this procedure creates a common skeleton based on the mean normalized FA image, which represents the core of the white matter tracts. All normalized FA, MD, AD and RD images were projected onto this skeleton using the projections factors. Mean DTI parameters were calculated within the skeleton.

#### 2.4.4 Structural network construction

To define network nodes, brain regions were segmented by the automated anatomical labelling (AAL) template, resulting in 45 regions for each hemisphere, excluding the cerebellum.(Tzourio-Mazoyer et al., 2002) Skull-stripped T1 images were non-linearly registered to the Montreal Neurological Institute (MNI) 152 template using Functional MRI of the Brain non-linear registration tool (FNIRT), part of the FSL 4.0.1 tools. The transformation matrix was derived from the registration of *b*0-images to T1 subject space using FLIRT and then used to register the AAL image to each subject's diffusion image space. To define network edges, we performed whole brain deterministic tractography. Fibre tracking was undertaken using Diffusion Toolkit (http://trackvis.org/dtk/). First, each voxel with a FA value >0.2 was seeded. Propagation of streamlines was estimated using the deterministic fibre assignment by continuous tracking (FACT) algorithm. This algorithm assumes uniform fibre orientation within voxels and sudden changes in fibre tract orientation at each voxels' boundaries. Streamlines ended if: (1) fibre tracks left the brain mask, (2) the tracking streamline encountered voxels with FA <0.2, or if (3) the turning angle exceeded 45°. A network edge (or connection) between two brain regions was assumed if the endpoints of a reconstructed streamline were located within both regions. Network edge was weighted as a function of the product of the number of streamlines between the brain regions and mean FA of the streamlines.(Tuladhar et al., 2016) We normalized edge weighting by the mean region volumes to correct for different sizes of the brain regions and different brain sizes. This procedure yielded an undirected, weighted 90 × 90 connectivity matrix for each individual participant.

#### 2.4.5 Graph theoretical analysis

We used the Brain Connectivity Toolbox to compute graph theoretical network measures.(Rubinov and Sporns, 2010) Calculated network measures included density of a network, defined as the total number of edges in a network divided by the possible number of edges, and average network strength, defined as the mean sum of all weighted edges for every node. To investigate the organisation of a network, we calculated the global and local efficiency. Global efficiency is the average inverse of the shortest path length, which is defined as the minimum number of weighted connections between two regions in a network. Local efficiency for a node is the global efficiency computed on first-degree neighbours of that node. Global efficiency is related to the extent of how well connected the brain regions are, while local efficiency is a measure of how well connected local clusters of brain regions are. To further characterise the constructed structural networks, we estimated the rich club coefficient and compared these between DM1 patients and unaffected controls.(van den Heuvel and Sporns, 2011) Rich clubs exist in a network, if certain nodes with a high degree, thus rich in connections, are also more densely interconnected among themselves than lower degree nodes in the network. We calculated the rich club coefficients across a range of degree as the ratio of the sum of the weighted connections between nodes of a certain degree and the sum of weights of the strongest connections in the total network. Next, we normalized the rich club coefficients, by dividing by the rich club coefficients calculated on a set of simulated random networks of equal size and degree distribution, to accommodate the fact that high degree nodes have a higher probability of being interconnected by chance alone. For these normalized coefficients, a rich club organisation was present if the normalized value was >1, thus larger than the coefficient calculated for the random networks. We designated connections between rich club nodes as rich club connections, connections to rich club nodes as feeder connections and connections between non-rich club nodes as peripheral connections. For each type of connection, we calculated the ‘connection strength’, a summary measure of connectivity, by summing the edge weights for that type and compared these between DM1 patients and controls.

### 2.5 Statistical analyses

We used SPSS (Version 22.0. Armonk, NY: IBM Corp.) for all statistical analysis. We evaluated normality using the Shapiro-Wilk test. Between group differences in demographics and neuropsychological performance were evaluated using Chi-Square tests, independent *t*-tests or Mann-Whitey *U* tests, as appropriate. For the neuropsychological evaluation, we controlled the false discovery rate using the Benjamini-Hochberg procedure, with false discovery rate Q set at 0.15.(MacDonald, 2018) For the MRI analyses, we used likelihood ratio tests for between-group comparisons of white matter hyperintensity load. We used analysis of univariate general linear modelling to evaluate differences in tissue volumes, whole-brain DTI parameters, and structural network measures, taking into account age, sex and education level. In the between-group structural network comparison, we evaluated the effect of grey matter volumes by adding them as a covariate in the linear models. For the TBSS analyses, we performed the voxel-wise group comparison between DM1 patients and unaffected control subjects, adjusted for age and sex, and voxel-wise associations between skeletal DTI parameters and cognitive outcome measures within DM1 patients, while adjusting for age, sex and education. For this, we applied permutation-based statistical interference tool for non-parametric approach (number of permutations is 5000) and significant clusters were identified using the threshold-free cluster enhancement with a *p*-value <.05, corrected for multiple comparisons.(Nichols and Holmes, 2002; Smith and Nichols, 2009) For the structural white matter network analysis, we applied network-based statistics (NBS) to investigate the location of network disruption.(Zalesky et al., 2010) A two-sample t-test was performed with t-value threshold of 2.4 (corresponding to *p* = .025 uncorrected), number of permutation testing set at 5000 and family-wise error (*p* < .05) to correct for multiple comparisons. Finally, we performed exploratory analyses to investigate the associations between neuropsychological, genetical and clinical measures with global DTI and network measures in the patient group. We utilised partial correlation, adjusting for age and education level into the model. With regards to neuropsychological test performance, partial correlations were only calculated for tests that showed significant between group differences. Alpha was set at 0.05 (two-sided) for these analyses.

### 2.6 Data availability

The data that have given rise to the results from this trial, are available on request via the corresponding author.

## 3 Results

### 3.1 Demographics and clinical characteristics

We included a total of 28 DM1 patients with a mean age of 46.0 [SD 9.0] and 26 age-matched unaffected controls with a mean age of 50.1 [SD 12.6] (Table 1). There were no statistically significant differences in estimated intelligence and the demographical parameters between groups. As expected, controls performed better on spirometry in comparison with DM1 patients (Table 1). For the MRI analysis, we additionally excluded three DM1 patients because of contraindications to MRI, a total of 25 DM1 patients underwent MRI. Of these 25 DM1 patients that underwent brain MRI, 16 and 9 had been in the standard care and behavioural intervention groups of the OPTIMISTIC study, respectively.

**Table 1:** Demographic and clinical parameters.

Variable	DM1 patients	Unaffected controls	P-value
Sex, no. of men/women	13/15	15/11	0.785
Age, mean in years ± SD	46.0 ± 9.0	50.2 ± 12.4	0.167
Educational level, mean ± SD	5.4 ± 1.0	5.4 ± 0.7	0.803
Functional vital capacity* (L), mean ± SD	3.3 (0.9)	4.4 (1.0)	0.0001
Estimated CTG progenitor allele length, mean ± SD ^	250.12 ± 140.57	N/A	N/A
Modal CTG repeat length, mean ± SD^	441.84 ± 210.33	N/A	N/A
Age at onset in years, mean ± SD^	24.1 ± 12.1	N/A	N/A
Clinical disease classification, no. (%) of patients with cDM1, iDM1, jDM1, aDM1, loDM1	0 (0), 1 (4), 12 (43), 12 (43), 2 (7)^#^	N/A	N/A
AES-c score^, mean ± SD	36.0 ± 7.9	N/A	N/A
DM1-activ-c^, mean ± SD	65.5 ± 15.7	N/A	N/A
CIS-fatigue^, mean ± SD	40.9 ± 7.5	N/A	N/A
MDHI^, mean ± SD	26.4 ± 15.0	N/A	N/A
MIRS score, no. (%) of patients^: 1/2/3/4/5	2 (7), 3 (11), 20 (71), 3 (11), 0 (0)	N/A	N/A
BDI-fs^ score, median [IQR]	2.0 [3.0]	N/A	N/A

^ Data collected in OPTIMISTIC main study; *sitting position. cDM1: congenital onset DM1; iDM1: infantile onset DM1, jDM1: juvenile onset DM1; aDM1 adult onset DM1; loDM1: late-onset DM1.(De Antonio et al., 2016) #for one patient, age at onset of symptoms was unknown. AES-c: apathy evaluation scale, clinician version, CIS-fatigue: checklist individual strength, fatigue subscale; MDHI: myotonic dystrophy health index; MIRS: muscular impairment rating scale; BDI-fs: Beck depression inventory fast-screen.

### 3.2 Neuropsychological assessment

The results of the neuropsychological assessment are presented in Table 2. DM1 patients performed worse than controls on the Stroop interference score (0.64 versus 0.70, *p* = .008), the d2 Test total score corrected for false answers (334.0 versus 405.5, *p* = .0005), the ROCF copy (30.0 versus 31.0, *p* = .037), ROCF immediate recall copy-corrected version (0.63 versus 0.74, *p* = .040) and the Pegboard Test (8.3 versus 10.0, *p* = .009). Controlling the false discovery rate using the Benjamini-Hochberg procedure, did not alter the significance of results.

**Table 2:** Neuropsychological test results in patients and controls: raw scores, *p*-values and effect sizes.

Cognitive test	Dir. of score	DM1 patients Mean (SD) or Median [IQR]	Unaffected controls Mean (SD) or Median [IQR]	P-Value^b^	Effect size^a^
NART-IQ	↑	97.2 (15.2)	98.7 (17.4)	0.741	0.1
RPM	↑	8.6 (2.0)	9.5 (1.7)	0.083	0.5
LM I	↑	31.0 [7.5]	27.5 [7.0]	0.202	0.3
LM II	↑	26.6 (6.1)	24.4 (6.2)	0.183	0.4
Category fluency (animals)	↑	27.4 (5.2)	25.9 (7.6)	0.400	0.2
Category fluency (occupations)	↑	21.2 (4.2)	20.9 (6.3)	0.839	0.1
TMT Interference score	↑	0.39 [0.11]	0.44 [0.15]	0.097	0.2
Stroop Color Word Test Interference score	↑	0.64 [0.10]	0.70 [0.10]	**0.008**^c^	0.7
Brixton Spatial Anticipation Test	↓	15.5 [5.5]	15.0 [9.0]	0.223	0.5
d2 test (Tn-F)	↑	334.0 [86.5]	405.5 [129.0]	**0.0005**^c^	1.0
ROCF copy	↑	30.0 [6.7]	31.0 [4.3]	**0.037**^c^	0.7
ROCF ir/copy	↑	0.63 [0.28]	0.74 [0.19]	**0.040**^c^	0.5
ROCF dr/copy	↑	0.65 [0.27]	0.75 [0.21]	0.100	0.4
Pegboard	↑	8.3 [5.5]	10.0 [2.5]	**0.009**^c^	0.9

dir: direction; NART: National adult reading test – IQ estimate; RPM: Raven's progressive matrices; LM I and LM II: Logical Memory of WSM-IV; ROCF: Rey-Osterrieth Complex Figure Test; IQR: interquartile range.

a Cohen's d is used as a measure of effect size, provided is the absolute effect size.

b Calculated using independent t-tests and non-parametric Mann-Whitney U tests for normally and non-normally distributed data. p-values <.05 are bold.

c All results remained significant after application of the Benjamini-Hochberg procedure with false-discovery rate Q set at 0.15.

### 3.3 MRI analyses

#### 3.3.1 White matter hyperintensities

DM1 patients had higher WMH load expressed as Fazekas scores in the periventricular and deep white matter in comparison to unaffected controls (Table 3). In contrast, no difference were found for WMH in the basal ganglia as determined by the ARWMC scale (Table 3).

**Table 3:** MRI analyses.

	DM1	Unaffected controls	*p*-value
White matter lesion load^a^	*N* = 24	*N* = 26	
Fazekas: periventricular white matter	2 ± 0.76	0.92 ± 0.56	p < .0001
mean ± SD, score 0/1/2/3	0 / 7 / 11 / 7	5 / 18 / 3 / 0	
Fazekas: deep white matter	1.16 ± 0.69	0.58 ± 0.50	*p* < .0001
mean ± SD, score 0/1/2/3	3 / 16 / 5 /1	11 / 15 / 0 / 0	
ARWMC	1.6 ± 0.65	0.54 ± 0.51	p < .0001
mean ± SD, score 0/1/2/3	0 / 12 / 11 / 2	12 / 14 / 0 / 0	
ARWMC basal ganglia	0.28 ± 0.61	0.19 ± 0.58	*p* = 0.539
mean ± SD, score 0/1/2/3	20 / 3 / 2 / 0	23 / 1 / 2 / 0	
Volumetry^b^	*N* = 23	*N* = 26	
Average grey matter volume – mean (SD)	619.8 (78.9)	690.6 (81.8)	p = 0.0005
Average white matter volume – mean (SD)	453.4 (58.4)	465.8 (61.9)	*p* = 0.611
Average CSF volume – mean (SD)	406.9 (91.7)	416.1 (62.4)	*p* = 0.660
Diffusion tensor imaging^b^	N = 23	N = 26	
FA – mean (SD)	0.50 (0.02)	0.56 (0.02)	p < 0.0001
MD – mean (SD)	0.08e^−2^ (0.03e^−3^)	0.07e^−2^ (0.03e^−3^)	p < 0.0001
Structural white matter networks^b^	*N* = 22	N = 22	
Density – mean (SD)	0.0985 (0.00537)	0.0973 (0.00335)	*p* = 0.312
Strength – mean (SD)	0.0383 (0.01238)	0.0509 (0.00887)	p < 0.0001
Global efficiency – mean (SD)	0.0023 (0.00072)	0.0031 (0.00053)	p < 0.0001
Local efficiency^c^−mean (SD)	0.0019 (0.00054)	0.0025 (0.00041)	*p* < 0.0001

White-matter hyperintensities in DM1 patients and control as measured with Fazekas and ARWMC scales.

a Likelihood ratio test.

b univariate GLM analysis p-value after correction for age, sex and educational level.

c Mean average across nodes.

#### 3.3.2 Tract-based spatial statistics

**Fig. 1:**
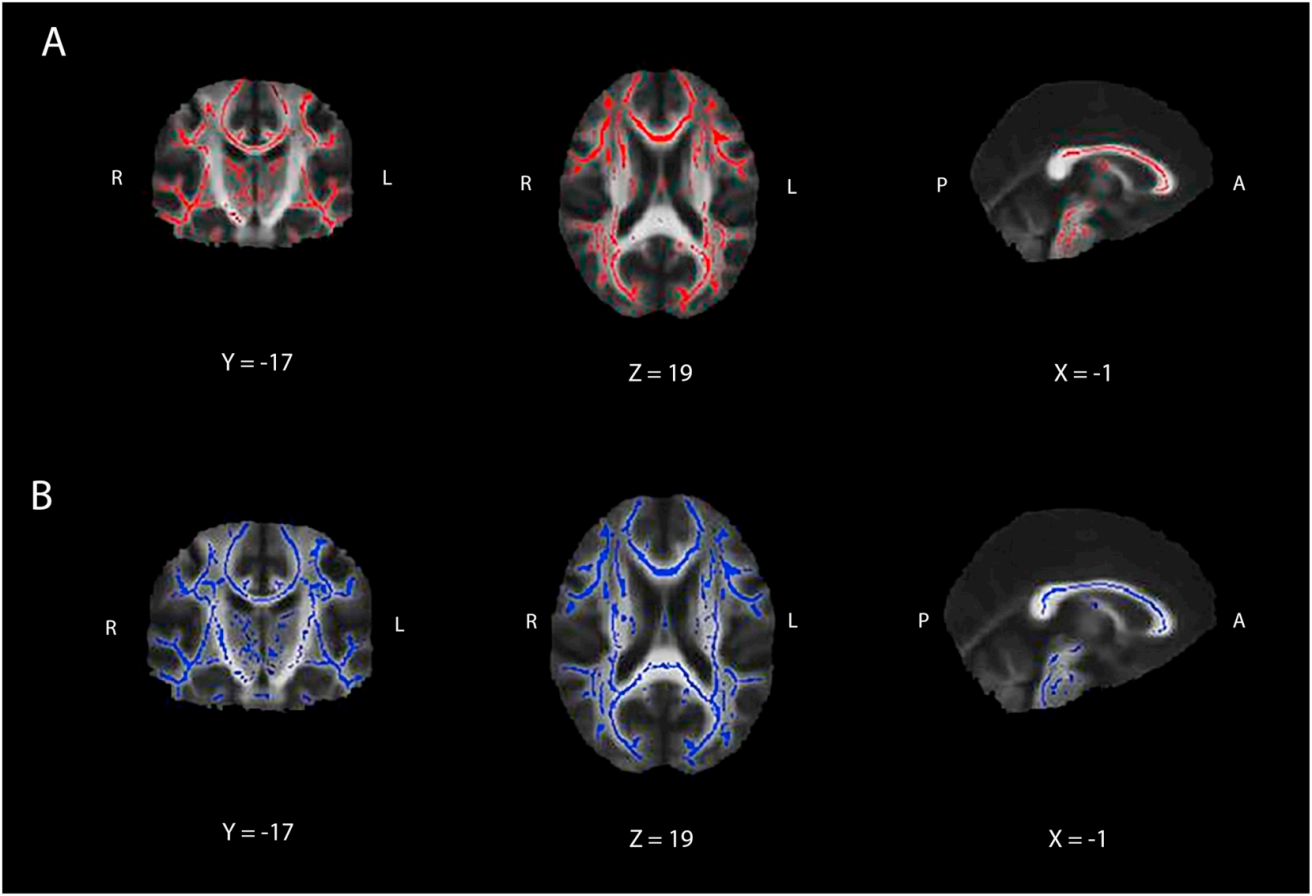
TBSS results. Differences for FA (red, A), MD (blue, B), between DM1 patients and unaffected controls. For both FA and MD, between-group differences can be seen widely dispersed throughout the brain and present in association, projection and commissural fibre systems (p < 0.05, corrected for multiple comparisons). (For interpretation of the references to color in this figure legend, the reader is referred to the web version of this article.)

We excluded two additional DM1 patients due to insufficient MRI quality for DTI analysis, thus resulting in 23 DM1 patients and 26 unaffected controls for the DTI analysis. Mean FA was significantly lower in DM1 patients compared with unaffected controls; mean MD was significantly higher in DM1 patients compared with healthy control (Table 3). As shown in Fig. 1, lower FA values and higher, AD and RD values were found in DM1 patients compared to unaffected controls and were widely dispersed throughout the brain, in projection, association and commissural fibre systems.

#### 3.3.3 Structural white matter network analyses

**Fig. 2:**
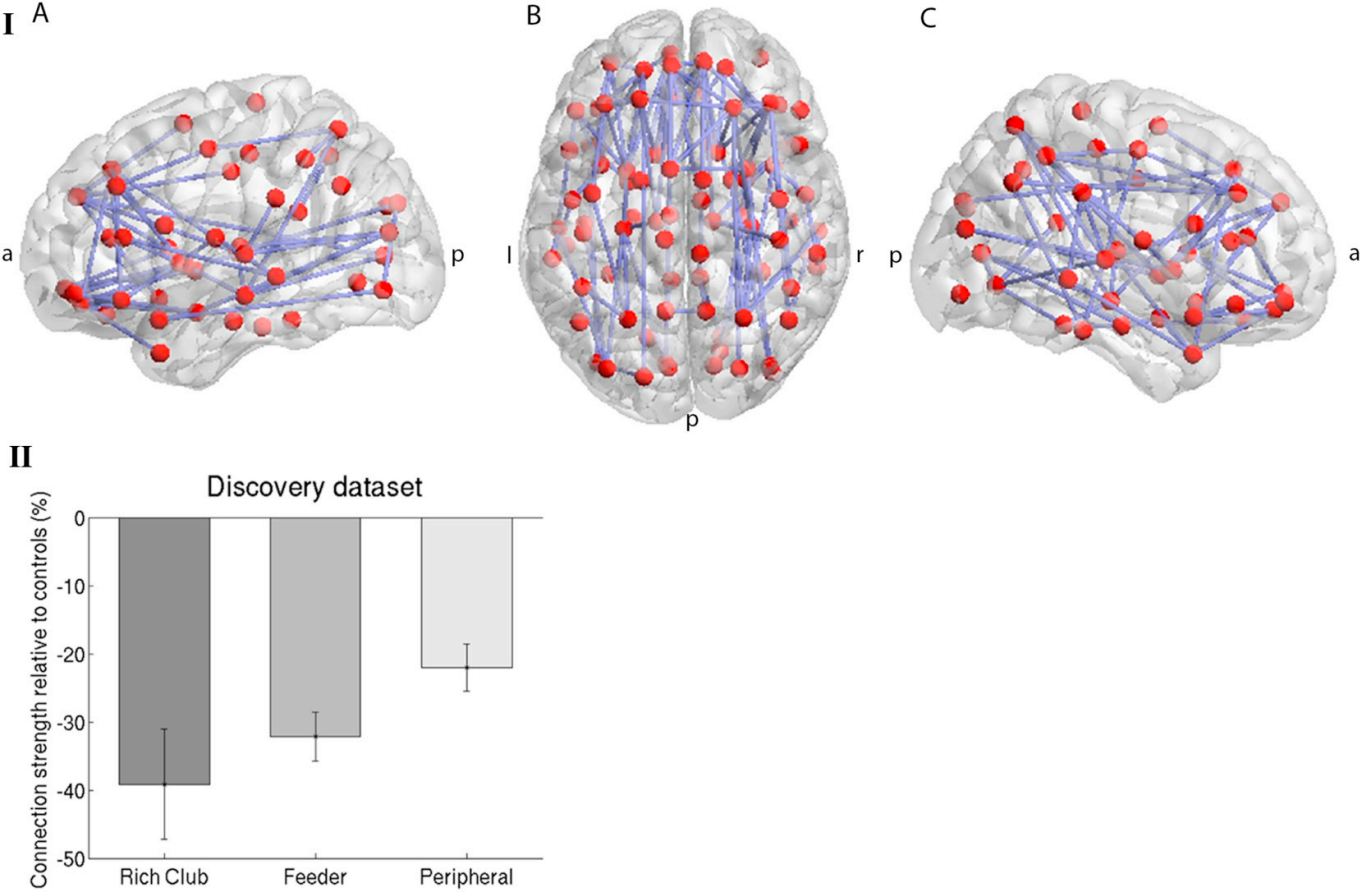
White matter networks in DM1 patients versus controls. I. Topological cluster that significantly differed between DM1 patients and unaffected controls (p-value adjusted <0.05, threshold *t* = 2.4 (corresponding with p-uncorrected 0.025). Networks were projected on the MNI152 standard space template, and visualized from the axial plane in neurological convention using the BrainNet Viewer toolbox in MATLAB.(Xia et al., 2013) a: anterior, p: posterior, r: right, l: left. Panel A: left lateral view. Panel B: Axial view from top. Panel C: right lateral view. II. Connection strength of rich club, feeder and peripheral connections of DM1 patients relative controls. Connections strength in DM1 patients was lower than in controls (see text); there were no statistically significant differences in connection strength between the 3 connection types within the DM1 patient group.

In graph theoretical analysis, the structural networks of DM1 patients showed lower network strength, lower global efficiency and lower local efficiency and a similar network density in comparison with controls (Table 3). These results were not altered if grey matter volumes were incorporated as a covariate in the analyses. In NBS analysis, we found a subnetwork of reduced connection strength in DM1 patients (*p* < .05) compared to controls (Fig. 2A). The reduced connections are diffusely located, in line with DTI results. Normalized rich club coefficients were >1 in DM1 and controls and reflect the presence of rich club organisation in both groups. Normalized rich club coefficients were however reduced in DM1 patients compared to controls (*p* < .001, 10.000 permutations). Group-wise analysis further indicated significantly reduced rich club (RC, *p* = .03), feeder (*p* < .0005) and peripheral connections (*p* = .001) in DM1 patients compared to unaffected controls. Although visually, there is a trend of more severe reduction in connection strength of rich club connections than feeder or peripheral connections (Fig. 2B), we found no statistically significant differences in connection strength between the different connection types within the DM1 patient group (RC – feeder connections (*p* = .50) and RC – peripheral connections (*p* = .11).

### 3.4 Correlation analyses

The results of exploratory, age and education corrected partial correlation analyses between whole brain FA, MD, global and local network efficiency and network strength, and neuropsychological tests that demonstrated significant between-group differences resulted in significant associations for the d2 Test and Purdue Pegboard Test, and the ROCF – copy subtest, with absolute correlation coefficients ranging from 0.458 to 0.554 (Table 4). No other significant correlations between DTI MRI parameters and neuropsychological performance were found. We found no significant correlations between DTI and structural network measures and clinical scales: CIS-fatigue and AES-c. No correlations were found between DTI and structural network measures and estimated CTG repeat expansion size (modal and estimated progenitor allele size). Finally, we found no significant associations between DTI and structural network measures and age at onset after adjustment for age and grey matter volume.

**Table 4:** Partial correlations between DTI and network parameters and neuropsychological performance in DM1 patients.

	FA	MD	Global network efficiency	Local network efficiency	Network strength
Stroop Interference score	0.070 (0.770)	−0.176 (0.459)	0.214 (0.365)	0.199 (0.401)	0.201 (0.395)
d2 Test	0.549 (0.012)*	−0.554 (0.011)*	0.535 (0.015)*	0.465(0.039)*	0.548 (0.012)*
ROCF - Copy	0.416 (0.068)	−0.475 (0.034)*	0.055 (0.817)	0.115 (0.628)	0.057 (0.811)
ROCF - Immediate recall (IR), copy corrected	−0.318 (0.172)	0.291 (0.213)	−0.167 (0.482)	−0.174 (0.463)	−0.195 (0.409)
Pegboard Test	0.549 (0.012)*	−0.404 (0.078)	0.462 (0.041)*	0.458 (0.042)*	0.482 (0.032)*

Depicted are the partial correlation coefficients (r) with the corresponding uncorrected *p*-values in parentheses. Correlation coefficients are corrected for age and level of education. FA: fractional anisotropy; MD: mean diffusivity; CWT: color-word test ROCF: Rey-Osterrieth Complex Figure.

## 4 Discussion

In this cross-sectional study, we demonstrate alterations in structural white matter networks in DM1 patients compared to unaffected controls. In comparison to unaffected controls, the networks in DM1 are characterised by reduced network strength combined with reduced global and local efficiency at a comparable network density. Thus, although the number of connections in the network of DM1 patients is not different from unaffected controls, the alterations in efficiency and strength are indicative of differences in structural network organisation. Although the presence of a rich-club (RC) organisation was present in both groups, normalized RC coefficients were reduced in DM1 patients in comparison to controls, signalling weaker connections between these nodes. A weaker connected rich club, the core of the connectome through which many network paths pass, may lead to a longer shortest path length overall within a network and hence, a network that is less integrated and less efficient at the global level.(Dennis et al., 2013) Furthermore, our results indicate that not only rich club connections, but also the feeder and peripheral connections have decreased strength in comparison with controls, indicating widespread structural alterations in the white matter networks. These diffuse network changes align with the diverse and anatomically unspecific structural changes in grey and white matter, such as grey matter volume reductions and alteration in white matter tract integrity in the current and in previous studies, as discussed below.(Antonini et al., 2004; Cabada et al., 2017)

This study on structural white matter networks may be considered in the context of four other DM1 studies evaluating MRI derived brain networks that were recently published (a structured summary of findings is given in Supplemental Table S1). Graph theoretical measures of global and local connectivity in structural grey matter networks in 28 DM1 patients were not different from 28 unaffected controls, although there were regional differences in anatomical hub distribution, indicating topologically different networks.(Sugiyama et al., 2017) Other DM1 MRI network studies in DM1 patients constitute functional resting-state MRI (rs-fMRI) studies, performed by one group of authors (Supplemental Table S1).(Serra et al., 2016a; Serra et al., 2016b; Serra et al., 2014) Graph theoretical network analyses of rs-fMRI did not reveal any changes in global network connectivity measures.(Serra et al., 2016a; Serra et al., 2016b) Both studies, carried out in partly overlapping cohorts, did report changes in local connectivity, such as nodal degree and efficiency, across different brain regions.(Serra et al., 2016a; Serra et al., 2016b) Although different data sources and analyses methods preclude direct comparisons, these and our network studies together demonstrate that the pathophysiological processes in DM1 consistently lead to changes in both structural and functional networks. They support the view of brain involvement in DM1 as ‘a complex network disorder’.

We hypothesized that DTI and structural network measures would be associated with neuropsychological performance, in particular of neuropsychological tests that are often abnormal in DM1 patients such as the ROCF-copy test.(Okkersen et al., 2017a; Sistiaga et al., 2010; Weber et al., 2010; Zalonis et al., 2010) In our study, out of the five tests that showed group effects, only the test for attention (d2 Test) and motor skill (Purdue Pegboard) are significantly associated with structural network measures. Notably, the correlation coefficients of network-based measures for these tests were not higher than those for the averaged whole-brain FA or MD values. Also, both tests are critically dependent on motor function that is expected to be compromised in DM1. The relative lack of associations between structural white matter network measures and other neuropsychological measures is remarkable, given that white matter network disruptions comparable to those observed in our study have been found to be associated with cognitive deficits in other disorders characterised by white matter damage, such as multiple sclerosis and cerebral small-vessel disease.(Lawrence et al., 2014; Llufriu et al., 2017; Tuladhar et al., 2016) More than one factor may contribute to the relative lack of associations between network measures and neuropsychological performance in our study. The differences in neuropsychological performance between the patient and control group are relatively small in our sample of DM1 patients. Despite diffuse alterations in brain structure, the brain in DM1 seems to be able to upkeep neuropsychological performance to some extent, possibly as a results of unknown compensatory mechanisms. Furthermore, functional network connectivity or decoupling between functional-structural connectivity, not assessed in this study, might be an important factor determining cognitive performance in DM1 patients.(Serra et al., 2016b) Finally, it may be explained by the small sample size and related statistical power issues in comparison to studies in other disorders. Similar reasons may explain the lack of associations between structural white matter network measures and measures of apathy and experienced fatigue.

In the ‘conventional’ MRI analysis, our results demonstrating increased white matter hyperintensity load and widespread alterations in white matter tract integrity corroborate earlier work and are indicative of widespread involvement of white matter tracts (Baldanzi et al., 2016; Minnerop et al., 2011; Zanigni et al., 2016). Possible neuropathological correlates of these white matter changes include loss of axons and/or myelin, capillary hyalinization and fibrillary gliosis, although these is scarce literature on human brain white matter histopathology in DM1.(Gourdon and Meola, 2017; Itoh et al., 2010) Future studies should preferably include in vivo white matter imaging combined with post-mortem histopathological observations.

This study has several limitations. We recruited DM1 patients that had previously participated in the OPTIMISTIC trial, who performed relatively well on the neuropsychological assessment, had relatively high levels of education, and were motivated to participate in clinical research. Consequently, the individuals in our study could be argued to be a relatively lesser cognitively affected sample of the DM1 population, as supported effect sizes on neuropsychological tests mostly smaller than those estimated in a recent meta-analysis.(Okkersen et al., 2017a) Generalisability of our findings may also be limited by the selection of DM1 patients in OPTIMISTIC that were severely fatigued as defined by a checklist individual strength fatigue subscale score of ≥35. However, severe fatigue as defined by this criterion occurs in 70% of the DM1 population.(Kalkman et al., 2005) Furthermore, because of the cross-sectional design of MRI sub-study, we cannot infer possible effects of the OPTIMISTIC behavioural intervention on structural white matter networks. Finally, we did not evaluate controls for depression of anxiety, although these symptoms may have been present as a result of a the emotional and social burden of a chronic disease of a family member or close friend.

The reader should note that TBSS, although a robust method for white matter analysis, may have a less than desired anatomical specificity. The use of the traditional ‘mean FA image’ method, which discards the directional information of the tensor in the skeleton construction step, may compromise the anatomical specificity of TBSS.(Bach et al., 2014) Construction of the FA skeleton may also be less alignment invariant than assumed and the projection step onto the FA skeleton may also vary in quality, especially in disease states such as DM1, where there are concurrent changes in brain morphology.(Bach et al., 2014; Keihaninejad et al., 2012) These limitations of TBSS necessitate cautious interpretation of results. With regards to the measures of the white matter structural network, these may be dependent on the fibre tracking algorithm and the choice of the anatomical labelling to define network nodes, as well as on network density.(Duda et al., 2014) It would be interesting to see if probabilistic and deterministic fibre tracking algorithms provide similar results on structural networks. Future research may include the cerebellum, which is of potential interest in DM1 (Minnerop et al., 2018); it was excluded in the current study as the deterministic tracking algorithm we utilised is potentially less accurate with cerebellar connections.Furthermore, network-based statistics may be influenced by the choice of the threshold, as explained previously.(Zalesky et al., 2010) Finally, the correlation analyses should be considered exploratory given the undertaking of a large number of statistical tests. The findings in the current study therefore warrant independent replication in further studies. Future imaging research in larger (multinational) clinically and/or genetically stratified DM1 cohorts with a prospective design could combine structural and functional imaging techniques and incorporate possible clinical correlates not evaluated here, such as personality traits and psychiatric symptoms.

## Funding

Funded by the European Community's Seventh Framework Programme (FP7/2007–2013; grant agreement n° 305697) and the Marigold Foundation. The funders of this trial had no role in the study design, data collection, analysis, interpretation of data, writing the report, or decisions regarding when to submit publications.

## Authors' competing interests

Ms. van Dorst has no competing interests to disclose.

Mr. Okkersen has no competing interests to disclose.

Dr. Meijer has no competing interests to disclose.

Professor Kessels has no competing interests to disclose.

Professor Monckton has been a paid scientific consultant and/or received an honoraria from Biogen Idec, AMO Pharma, Charles River and Vertex Pharmaceuticals. Professor Monckton also had a research contract with AMO Pharma and has received awards from the European Union, Muscular Dystrophy UK and the Myotonic Dystrophy Support Group.

Professor Van Engelen reports grant support from the European Union FP-7 program (OPTIMISTIC) and Marigold Foundation.

Dr. Tuladhar reports grants from Junior Staff Member Dutch Heart Foundation 2016 T044.

Dr. Raaphorst reports grant support from the Marigold Foundation.

## Author contributions

This study was conceptualized by KO, JR and AT. All authors were involved in the design of the study and preparation of the manuscript. Statistical analysis performed by MD and AT. The first and second authors wrote the first draft of this manuscript. All authors attest to the completeness and accuracy of data and corresponding analyses.

## External Advisory board

Dr. Marie Kierkegaard (Karolinska Instituet Medical University, Huddinge, Sweden).

## Authors' competing interests

Ms. van Dorst has no competing interests to disclose; Mr. Okkersen has no competing interests to disclose; Dr. Meijer has no competing interests to disclose; Professor Kessels has no competing interests to disclose; Professor Monckton has been a paid scientific consultant and/or received an honoraria from Biogen Idec, AMO Pharma, Charles River and Vertex Pharmaceuticals. Professor Monckton also had a research contract with AMO Pharma and has received awards from the European Union, Muscular Dystrophy UK and the Myotonic Dystrophy Support Group; Professor Van Engelen reports grant support from the European Union FP-7 program (OPTIMISTIC) and Marigold Foundation; Dr. Tuladhar reports grants from Junior Staff Member Dutch Heart Foundation 2016 T044; Dr. Raaphorst reports grant support from the Marigold Foundation.

## Appendix A Supplementary data

Supplementary material Image 1

## References

[bb0005] Antonini G., Mainero C., Romano A., Giubilei F., Ceschin V., Gragnani F., Morino S., Fiorelli M., Soscia F., Di Pasquale A., Caramia F. (2004). Cerebral atrophy in myotonic dystrophy: a voxel based morphometric study. J. Neurol. Neurosurg. Psychiatry.

[bb0010] Antonini G., Soscia F., Giubilei F., De Carolis A., Gragnani F., Morino S., Ruberto A., Tatarelli R. (2006). Health-related quality of life in myotonic dystrophy type 1 and its relationship with cognitive and emotional functioning. J. Rehabil. Med..

[bb0015] Bach M., Laun F.B., Leemans A., Tax C.M., Biessels G.J., Stieltjes B., Maier-Hein K.H. (2014). Methodological considerations on tract-based spatial statistics (TBSS). NeuroImage.

[bb0020] Baldanzi S., Cecchi P., Fabbri S., Pesaresi I., Simoncini C., Angelini C., Bonuccelli U., Cosottini M., Siciliano G. (2016). Relationship between neuropsychological impairment and grey and white matter changes in adult-onset myotonic dystrophy type 1. Neuroimage Clin..

[bb0025] Bird T.D., Pagon R.A., Adam M.P., Ardinger H.H., Wallace S.E., Amemiya A., Bean L.J.H., Bird T.D., Dolan C.R., Fong C.T., Smith R.J.H., Stephens K. (1993). Myotonic dystrophy type 1. GeneReviews(R).

[bb0030] Bressler S.L., Menon V. (2010). Large-scale brain networks in cognition: emerging methods and principles. Trends Cogn. Sci..

[bb0035] Bullmore E., Sporns O. (2009). Complex brain networks: graph theoretical analysis of structural and functional systems. Nat. Rev. Neurosci..

[bb0040] Cabada T., Iridoy M., Jerico I., Lecumberri P., Seijas R., Gargallo A., Gomez M. (2017). Brain involvement in myotonic dystrophy type 1: a Morphometric and diffusion tensor imaging study with neuropsychological correlation. Arch. Clin. Neuropsychol..

[bb0045] De Antonio M., Dogan C., Hamroun D., Mati M., Zerrouki S., Eymard B., Katsahian S., Bassez G., French Myotonic Dystrophy Clinical (2016). Unravelling the myotonic dystrophy type 1 clinical spectrum: a systematic registry-based study with implications for disease classification. Rev. Neurol. (Paris).

[bb0050] Dennis E.L., Jahanshad N., Toga A.W., McMahon K.L., de Zubicaray G.I., Hickie I., Wright M.J., Thompson P.M. (2013). Development of the "Rich Club" in brain connectivity networks from 438 adolescents & adults aged 12 to 30. Proc. IEEE Int Symp Biomed Imaging.

[bb0055] Duda J.T., Cook P.A., Gee J.C. (2014). Reproducibility of graph metrics of human brain structural networks. Front Neuroinform.

[bb0060] Duits A., Kessels R., Hendriks M., Kessels R., Gorissen M., Schmand B., Duits A. (2014). Schatten van het premorbide functioneren [estimating premorbid intelligence]. Neuropsychologische Diagnostiek: de klinische praktijk.

[bb0065] Fazekas F., Chawluk J.B., Alavi A., Hurtig H.I., Zimmerman R.A. (1987). MR signal abnormalities at 1.5 T in Alzheimer's dementia and normal aging. AJR Am. J. Roentgenol..

[bb0070] Gagnon C., Mathieu J., Jean S., Laberge L., Perron M., Veillette S., Richer L., Noreau L. (2008). Predictors of disrupted social participation in myotonic dystrophy type 1. Arch. Phys. Med. Rehabil..

[bb0075] Gourdon G., Meola G. (2017). Myotonic Dystrophies: State of the Art of New Therapeutic Developments for the CNS. Front. Cell. Neurosci..

[bb0080] Harper P.S. (2001). Myotonic Dystrophy.

[bb0085] Itoh K., Mitani M., Kawamoto K., Futamura N., Funakawa I., Jinnai K., Fushiki S. (2010). Neuropathology does not Correlate with Regional differences in the Extent of expansion of CTG Repeats in the Brain with Myotonic Dystrophy Type 1. Acta Histochem. Cytochem..

[bb0090] Kalkman J.S., Schillings M.L., van der Werf S.P., Padberg G.W., Zwarts M.J., van Engelen B.G., Bleijenberg G. (2005). Experienced fatigue in facioscapulohumeral dystrophy, myotonic dystrophy, and HMSN-I. J. Neurol. Neurosurg. Psychiatry.

[bb0095] Keihaninejad S., Ryan N.S., Malone I.B., Modat M., Cash D., Ridgway G.R., Zhang H., Fox N.C., Ourselin S. (2012). The importance of group-wise registration in tract based spatial statistics study of neurodegeneration: a simulation study in Alzheimer's disease. PLoS One.

[bb0100] Laberge L., Mathieu J., Auclair J., Gagnon E., Noreau L., Gagnon C. (2013). Clinical, psychosocial, and central correlates of quality of life in myotonic dystrophy type 1 patients. Eur. Neurol..

[bb0105] Lawrence A.J., Chung A.W., Morris R.G., Markus H.S., Barrick T.R. (2014). Structural network efficiency is associated with cognitive impairment in small-vessel disease. Neurology.

[bb0110] Lezak M.D. (2012). Neuropsychological Assessment.

[bb0115] Llufriu S., Martinez-Heras E., Solana E., Sola-Valls N., Sepulveda M., Blanco Y., Martinez-Lapiscina E.H., Andorra M., Villoslada P., Prats-Galino A., Saiz A. (2017). Structural networks involved in attention and executive functions in multiple sclerosis. Neuroimage Clin.

[bb0120] MacDonald J.H. (2018). Handbook of Biological Statistics.

[bb0125] Minnerop M., Weber B., Schoene-Bake J.C., Roeske S., Mirbach S., Anspach C., Schneider-Gold C., Betz R.C., Helmstaedter C., Tittgemeyer M., Klockgether T., Kornblum C. (2011). The brain in myotonic dystrophy 1 and 2: evidence for a predominant white matter disease. Brain.

[bb0130] Minnerop M., Gliem C., Kornblum C. (2018). Current Progress in CNS Imaging of Myotonic Dystrophy. Front. Neurol..

[bb0135] Nichols T.E., Holmes A.P. (2002). Nonparametric permutation tests for functional neuroimaging: a primer with examples. Hum. Brain Mapp..

[bb0140] Okkersen K., Buskes M., Groenewoud J., Kessels R.P.C., Knoop H., van Engelen B., Raaphorst J. (2017). The cognitive profile of myotonic dystrophy type 1: a systematic review and meta-analysis. Cortex.

[bb0145] Okkersen K., Monckton D.G., Le N., Tuladhar A.M., Raaphorst J., van Engelen B.G.M. (2017). Brain imaging in myotonic dystrophy type 1: a systematic review. Neurology.

[bb0150] Okkersen K., Jimenez-Moreno C., Wenninger S., Daidj F., Glennon J., Cumming S., Littleford R., Monckton D.G., Lochmuller H., Catt M., Faber C.G., Hapca A., Donnan P.T., Gorman G., Bassez G., Schoser B., Knoop H., Treweek S., van Engelen B.G.M., Consortium O. (2018). Cognitive behavioural therapy with optional graded exercise therapy in patients with severe fatigue with myotonic dystrophy type 1: a multicentre, single-blind, randomised trial. Lancet Neurol..

[bb0155] Oosterman J.M., Vogels R.L., van Harten B., Gouw A.A., Poggesi A., Scheltens P., Kessels R.P., Scherder E.J. (2010). Assessing mental flexibility: neuroanatomical and neuropsychological correlates of the Trail making Test in elderly people. Clin. Neuropsychol..

[bb0160] Raaphorst J., de Visser M., van Tol M.J., Linssen W.H., van der Kooi A.J., de Haan R.J., van den Berg L.H., Schmand B. (2011). Cognitive dysfunction in lower motor neuron disease: executive and memory deficits in progressive muscular atrophy. J. Neurol. Neurosurg. Psychiatry.

[bb0165] Rubinov M., Sporns O. (2010). Complex network measures of brain connectivity: uses and interpretations. NeuroImage.

[bb0170] Schaapsmeerders P., Maaijwee N.A., van Dijk E.J., Rutten-Jacobs L.C., Arntz R.M., Schoonderwaldt H.C., Dorresteijn L.D., Kessels R.P., de Leeuw F.E. (2013). Long-term cognitive impairment after first-ever ischemic stroke in young adults. Stroke.

[bb0175] Serra L., Silvestri G., Petrucci A., Basile B., Masciullo M., Makovac E., Torso M., Spano B., Mastropasqua C., Harrison N.A., Bianchi M.L., Giacanelli M., Caltagirone C., Cercignani M., Bozzali M. (2014). Abnormal functional brain connectivity and personality traits in myotonic dystrophy type 1. JAMA Neurol.

[bb0180] Serra L., Cercignani M., Bruschini M., Cipolotti L., Mancini M., Silvestri G., Petrucci A., Bucci E., Antonini G., Licchelli L., Spano B., Giacanelli M., Caltagirone C., Meola G., Bozzali M. (2016). "I know that you know that I know": Neural Substrates Associated with Social Cognition Deficits in DM1 patients. PLoS One.

[bb0185] Serra L., Mancini M., Silvestri G., Petrucci A., Masciullo M., Spano B., Torso M., Mastropasqua C., Giacanelli M., Caltagirone C., Cercignani M., Meola G., Bozzali M. (2016). Brain Connectomics' Modification to Clarify Motor and Nonmotor Features of Myotonic Dystrophy Type 1. Neural Plast.

[bb0190] Shin M.S., Park S.Y., Park S.R., Seol S.H., Kwon J.S. (2006). Clinical and empirical applications of the Rey-Osterrieth complex Figure Test. Nat. Protoc..

[bb0195] Sistiaga A., Urreta I., Jodar M., Cobo A.M., Emparanza J., Otaegui D., Poza J.J., Merino J.J., Imaz H., Marti-Masso J.F., Lopez De Munain A. (2010). Cognitive/personality pattern and triplet expansion size in adult myotonic dystrophy type 1 (DM1): CTG repeats, cognition and personality in DM1. Psychol. Med..

[bb0200] Smith S.M., Nichols T.E. (2009). Threshold-free cluster enhancement: addressing problems of smoothing, threshold dependence and localisation in cluster inference. NeuroImage.

[bb0205] Smith S.M., Jenkinson M., Johansen-Berg H., Rueckert D., Nichols T.E., MacKay C.E., Watkins K.E., Ciccarelli O., Cader M.Z., Matthews P.M., Behrens T.E. (2006). Tract-based spatial statistics: voxelwise analysis of multi-subject diffusion data. NeuroImage.

[bb0210] Stam C.J. (2014). Modern network science of neurological disorders. Nat. Rev. Neurosci..

[bb0215] Sugiyama A., Sone D., Sato N., Kimura Y., Ota M., Maikusa N., Maekawa T., Enokizono M., Mori-Yoshimura M., Ohya Y., Kuwabara S., Matsuda H. (2017). Brain gray matter structural network in myotonic dystrophy type 1. PLoS One.

[bb0220] Tiffin J., Asher E.J. (1948). The Purdue pegboard; norms and studies of reliability and validity. J. Appl. Psychol..

[bb0225] Tuladhar A.M., van Dijk E., Zwiers M.P., van Norden A.G., de Laat K.F., Shumskaya E., Norris D.G., de Leeuw F.E. (2016). Structural network connectivity and cognition in cerebral small vessel disease. Hum. Brain Mapp..

[bb0230] Tzourio-Mazoyer N., Landeau B., Papathanassiou D., Crivello F., Etard O., Delcroix N., Mazoyer B., Joliot M. (2002). Automated anatomical labeling of activations in SPM using a macroscopic anatomical parcellation of the MNI MRI single-subject brain. NeuroImage.

[bb0235] van den Heuvel M.P., Sporns O. (2011). Rich-club organization of the human connectome. J. Neurosci..

[bb0240] van Engelen B., Consortium O. (2015). Cognitive behaviour therapy plus aerobic exercise training to increase activity in patients with myotonic dystrophy type 1 (DM1) compared to usual care (OPTIMISTIC): study protocol for randomised controlled trial. Trials.

[bb0245] Wahlund L.O., Barkhof F., Fazekas F., Bronge L., Augustin M., Sjogren M., Wallin A., Ader H., Leys D., Pantoni L., Pasquier F., Erkinjuntti T., Scheltens P., European Task Force on Age-Related White Matter (2001). A new rating scale for age-related white matter changes applicable to MRI and CT. Stroke.

[bb0250] Wang Z., Dai Z., Gong G., Zhou C., He Y. (2015). Understanding structural-functional relationships in the human brain: a large-scale network perspective. Neuroscientist.

[bb0255] Weber Y.G., Roebling R., Kassubek J., Hoffmann S., Rosenbohm A., Wolf M., Steinbach P., Jurkat-Rott K., Walter H., Reske S.N., Lehmann-Horn F., Mottaghy F.M., Lerche H. (2010). Comparative analysis of brain structure, metabolism, and cognition in myotonic dystrophy 1 and 2. Neurology.

[bb0260] Xia M., Wang J., He Y. (2013). BrainNet Viewer: a network visualization tool for human brain connectomics. PLoS One.

[bb0265] Yan C., Gong G., Wang J., Wang D., Liu D., Zhu C., Chen Z.J., Evans A., Zang Y., He Y. (2011). Sex- and brain size-related small-world structural cortical networks in young adults: a DTI tractography study. Cereb. Cortex.

[bb0270] Zalesky A., Fornito A., Bullmore E.T. (2010). Network-based statistic: identifying differences in brain networks. NeuroImage.

[bb0275] Zalesky A., Fornito A., Seal M.L., Cocchi L., Westin C.F., Bullmore E.T., Egan G.F., Pantelis C. (2011). Disrupted axonal fiber connectivity in schizophrenia. Biol. Psychiatry.

[bb0280] Zalonis I., Bonakis A., Christidi F., Vagiakis E., Papageorgiou S.G., Kalfakis N., Manta P., Vassilopoulos D. (2010). Toward understanding cognitive impairment in patients with myotonic dystrophy type 1. Arch. Clin. Neuropsychol..

[bb0285] Zanigni S., Evangelisti S., Giannoccaro M.P., Oppi F., Poda R., Giorgio A., Testa C., Manners D.N., Avoni P., Gramegna L.L., De Stefano N., Lodi R., Tonon C., Liguori R. (2016). Relationship of white and gray matter abnormalities to clinical and genetic features in myotonic dystrophy type 1. Neuroimage Clin..

[bb0290] Zwiers M.P. (2010). Patching cardiac and head motion artefacts in diffusion-weighted images. NeuroImage.

