# Supplementary material for: Structural white matter networks in myotonic dystrophy type 1
Source: Neuroimage Clin. 2018 Nov 28;21:101615. doi: 10.1016/j.nicl.2018.101615 (PMC6413352; doi:10.1016/j.nicl.2018.101615)
Supplement: Supplementary file 1 — Supplementary material [file mmc1.docx]

**Appendix**

**A.**

**Data security**

Hard-copy data from history, spirometry and neuropsychological assessment were anonymised, coded and safely stored at an appropriate data storage location. Data were then transferred to Castor, an electronic record database designed for research purposes, to facilitate data analysis. After generation, MRI images were stored locally at a secured server. Before data analysis, image data was anonymised and coded.

**B.**

**Supplemental Table S1** Network imaging studies in myotonic dystrophy type 1

| **Table S1.** | | | | | |
| --- | --- | --- | --- | --- | --- |
| **Author (study year)** | **No. of patients/ controls** | **Technique** | **Network based statistics** | **Graph theoretical analysis** | **Correlations** |
| Serra (2016) | 31/26 | RS-fMRI | ↓ overall connectivity, ↓ hub connectivity anterior cingulum, orbitofrontal cortex, right parahippocampal gyrus, ↓ peripheral node connectivity prefrontal, temporal, parietal and cerebellar | Global network connectivity: not different from controls  Local network connectivity LC - nodal degree: ↓ superior frontal gyrus, orbitofrontal gyrus ↑ SMA, cerebellum LC - betweenness centrality: ↓ right superior frontal gyrus, right inferior parietal gyrus, right putamen ↑ right paracentral lobule, cerebellum LC - Nodal efficiency: ↓right superior frontal gyrus, right orbitofrontal cortex, left angular gyrus | cognitive performance, (RPM) clinical parameters (MIRS, CTG repeat expansion) |
| Sugiyama (2017) | 28/28 | structural MRI (grey matter volumes) |  | No differences in global and local network properties (small-world-ness, characteristic path length, efficiency, modularity, transitivity, clustering). No differences in resilience between "random" and "targeted" attack. Different regional hub distributions between DM1 patients and controls |  |
| Serra (2016) | 20/18 | RS-fMRI | used to construct a theory-of-mind network | Global network connectivity: not different from controls. Local network connectivity: ↑ nodal efficiency and degree in left inferior temporal gyrus | theory-of-mind network and social cognition |
| Serra (2014) | 27/16 | RS-fMRI | Independent component analysis: increased DMN functional connectivity bilateral posterior cingulate and left parietal node |  | default mode network and personality |

**Caption table S1**

MIRS: muscular impairment rating scale; RPM: Raven’s progressive matrices; RS-fMRI: resting state functional MRI
